# Detection of cutaneous malignant melanoma using RNA sampled by tape strips: A study protocol

**DOI:** 10.1371/journal.pone.0274413

**Published:** 2022-09-21

**Authors:** Ida M. Heerfordt, Jeppe D. Andersen, Peter A. Philipsen, Linnea Langhans, Torben Tvedebrink, Grethe Schmidt, Thomas Poulsen, Catharina M. Lerche, Niels Morling, Hans Christian Wulf

**Affiliations:** 1 Department of Dermatology, Copenhagen University Hospital—Bispebjerg and Frederiksberg, Copenhagen, Denmark; 2 Department of Forensic Medicine, Section of Forensic Genetics, Faculty of Health and Medical Sciences, University of Copenhagen, Copenhagen, Denmark; 3 Department of Plastic Surgery and Burns Treatment, Copenhagen University Hospital—Rigshospitalet, Copenhagen, Denmark; 4 Department of Mathematical Sciences, Aalborg University, Aalborg, Denmark; 5 Department of Pathology, Hospital of Southern Jutland, Soenderborg, Denmark; 6 Department of Pharmacy, University of Copenhagen, Copenhagen, Denmark; University of Helsinki: Helsingin Yliopisto, FINLAND

## Abstract

**Background:**

Cutaneous malignant melanoma (CMM) is curable if detected in its early stages. However, the clinical recognition of CMM is challenging. An American research group has shown promising results in detecting CMM based on RNA profiles sampled from suspicious lesions with tape strips. We aim to further develop this technique and validate if RNA profiles sampled with tape strips can detect CMM.

**Methods:**

This prospective cohort study will include approximately 200 lesions clinically suspected of CMM requiring surgical removal. Tape stripping of the lesions will be performed just before surgical excision. Subsequently, RNA on the tape strips is analyzed using quantitative real-time polymerase chain reaction with TaqMan technology. The results are combined into a binary outcome where positive indicates CMM and negative indicates no CMM. The histopathological diagnosis of the lesions will be used as the gold standard. The main outcome is the results of the RNA test and the histopathological diagnosis, which, combined, provide the sensitivity and specificity of the test.

**Discussion:**

The accuracy of the clinical examination in CMM diagnostics is limited. This clinical trial will explore the ability to use RNA analysis to improve the management of suspicious lesions by enhancing early diagnostic accuracy. Hopefully, it can reduce the number of benign lesions being surgically removed to rule out CMM and decrease patient morbidity.

**Trial registration:**

The project was approved by The Committee on Health Research Ethics of the Capital Region of Denmark (H-15010559) and registered at the Danish Data Protection Agency (BFH-2015-065).

## Introduction

Worldwide, about 60,000 individuals die from cutaneous malignant melanoma (CMM) each year [1]. CMM is curable if detected in its early stages, but the diagnostic accuracy of the clinical examination is limited [2–4]. A CMM diagnosis established late in the disease progression results in poor treatment outcomes [4]. On the other hand, many benign lesions are surgically removed to exclude CMM [2]. If a simple and reliable method for the detection of CMM is made available, CMM might be diagnosed earlier, thereby improving treatment. Genetic alterations and changes in gene expression profiles are important early steps in melanoma pathogenesis [5–7]. Gene expression can be investigated by the examination of RNA profiles [7].

An American research group supported by DermTech (La Jolla, CA, USA) has proposed a non-invasive RNA test to improve the accuracy of CMM diagnostics using RNA profiles sampled with tape strips from the surface of suspicious lesions [8–13]. The RNA test is presented as a rule-out test [14]. An overview of the different study protocols used by the American research group is presented in Table 1. Several details in the protocols are not publicly accessible. An early version of their test investigated tape strips for levels of 17 RNAs corresponding to the expression profiles of 17 genes presented by Wachsman et al. [8] This test had a sensitivity of 100% and a specificity of 88% [8]. Later, the RNA test was changed to examine the expression of only two genes, *LINC00518* and *CMIP* [9]. In 2017, the two genes were switched to *LINC00518* and *PRAME*, which the group uses in their commercially available test named “pigmented lesion assay” [10, 11]. *LINC00518* and *PRAME* are known to be overexpressed in CMM, and the test result is deemed positive (indication CMM) or negative (indicating absence of CMM) based on the detection of RNA from one or both genes. The test has a reported sensitivity between 91% and 95% and a specificity between 69% and 91% [10, 12, 13]. The sensitivity level, in particular, has raised concerns about the strength of the RNA test and has resulted in a need for further improvement and validation of the test methods [15, 16]. We believe that the sensitivity of the RNA test must be close to 100% to be widely accepted and used. Consequently, we included more than two genes (see Table 2) in this study. We aim to further develop this technique and validate if RNA profiles sampled with tape strips can be used to detect CMM among suspicious lesions.

**Table 1:** Study protocols.

Study	Minimum size of the lesion	Type of tape	TS per lesion	TS storage time	TS storage temperature	RNA quantification	Disease related RNAs tested, N	Housekeeping genes tested, N	Reference diagnoses	Tested lesions, N	RNA test sensitivity	RNA test specificity
Wachsman et al. [8]	4 mm in diameter	Custom made	4	NR	NR	TaqMan qRT-PCR	17	1	HE	128	100%	88%
Gerami et al. [9]	4 mm in diameter	NR	4	Maximum 1 week	Minus 20°C	TaqMan qRT-PCR	2	3	HE	64	98%	73%
Gerami et al. [10]	4 mm in diameter	Adhesive patch (DermTech Inc, La Jolla, CA, USA)	4	NR	Minus 80°C	TaqMan qRT-PCR	2	1	HE	398	91%	69%
Ferris et al. [12]	NR	Adhesive patch (DermTech Inc, La Jolla, CA, USA)	4	NR	NR	NR	2	NR	HE in 55 cases Clinical follow-up in 326 cases	381	95%	91%
Heerfordt et al. The present protocol	Longest diameter 3 mm	Sterile hospital tape (Mölnlycke Health Care, Göteborg, Sweden)	2	Up to one month	Room temperature. Dry and dark	TaqMan qRT-PCR	9	2	HE	≈200	?	?

Overview of different study protocols for detecting cutaneous malignant melanoma (CMM) using RNA analysis of tape strips. Abbreviations: HE: histopathological examination, N: number, NR: not reported, TS: tape strip, qRT-PCR: quantitative real-time polymerase chain reaction.

**Table 2:** List of the 11 genes to be tested in the present study with procedures and justifications for testing them.

Gene name (HGNC ID)	TaqMan probe number	Volume of RNA solution for qRT-PCR	Biological function	Justification
*CMIP* (24319)	Hs01585625_g1	4.0 μl	Plays a role in T cell signaling pathway	Downregulated in CMM [8, 9]
*CNN2* (2156)	Hs00854264_s1	2.0 μl	Regulator of cell proliferation and apoptosis	Downregulated in CMM [8]
*GPM6B* (4461)	Hs01041077_m1	3.0 μl	Involved in melanogenesis	Upregulated in CMM [8]
*KIT* (6342)	Hs00174029_m1	7.0 μl	Regulator of cell proliferation and melanogenesis	Upregulated in CMM [8]
*LINC00518* (28626)	Hs00991460_g1	4.0 μl	Influences cell proliferation	Upregulated in CMM [8–10]
*PRAME* (9336)	Hs01022301_m1	7.0 μl	Regulator of cell proliferation and apoptosis	Upregulated in CMM [8, 10]
*RPL18* (10310)	Hs00965812_g1	2.5 μl	Ribosomal protein important for the regulation of cell proliferation	Downregulated in CMM [8]
*RPL21* (10313)	Hs03003806_g1	2.0 μl	Ribosomal protein important for the regulation of cell proliferation	Downregulated in CMM [8]
*RPS15* (10388)	Hs01358643_g1	2.5 μl	Ribosomal protein important for the regulation of cell proliferation	Downregulated in CMM [8]
*GAPDH* (4141)	Hs02758991_g1	3.0 μl	Key enzyme in glycolysis	Housekeeping gene
*CLTC* (2092)	Hs00964504_m1	3.0 μl	Protein component of organelles involved in intracellular trafficking	Housekeeping gene

The genes are investigated through the level of their corresponding RNA sequences. Abbreviations: CMM: cutaneous malignant melanoma, HGNC ID: Unique gene number provided by the HUGO Gene Nomenclature Committee, qRT-PCR: quantitative real-time polymerase chain reaction.

We established the RNA analysis method described in the Methods section, which makes it possible to quantify RNA profiles sampled noninvasively with tape. Here, sufficient material on the tape strips is collected to examine the expression profiles of up to 11 genes. To be able to correct for the size of the lesions and amount of collected material, we examine expressions of two housekeeping genes, *GAPDH* and *CLTC*, leaving room for nine disease related RNAs, which we chose from the 17 RNA markers first proposed by Wachsman et al. [8] The 11 selected RNAs are presented with justifications in Table 2. The results of the RNA profiles will be combined into a binary outcome where positive indicates CMM and negative indicates the absence of CMM.

## Methods

### Design and objectives

**Fig 1:**
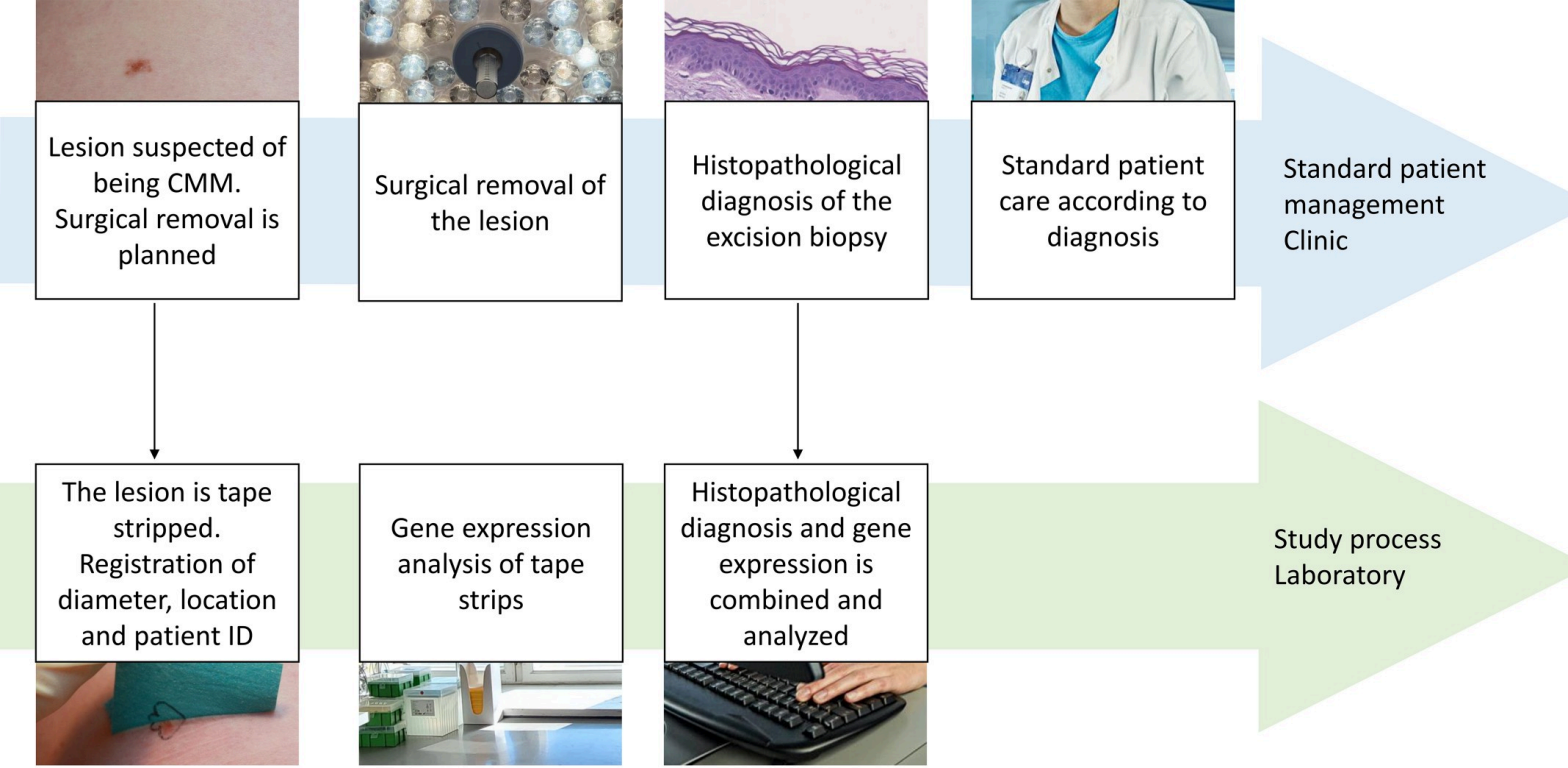
Study process. Overview of the standard patient management and study process initiated after obtaining written informed consent. During the study process, gene expression is investigated through RNA profiles on tape strips. The two black arrows indicate the transfer of data or material from the clinic to the project. No data is transferred from the project to the clinic. Clinical patient management is unaffected by the study and takes place without delay. Abbreviations: CMM: cutaneous malignant melanoma, patient ID: unique Danish personal identification number.

The study is an observational prospective cohort study and does not affect the patients’ treatment which follows standard guidelines. An overview of the study process is provided in Fig 1. The main aim of the study is to investigate whether RNA profiles sampled with tape strips can detect CMM among suspicious lesions. The primary outcome is the sensitivity and specificity of the RNA test. Secondary outcomes are the impact on the RNA profiles of patient age, lesion diameter, skin area involved, and storage time of the tape before analysis. The findings of this study will be published in peer-reviewed journals and presented at relevant scientific conferences. A separate purpose of this study protocol is to make tape strip method details publicly available.

### Participant eligibility criteria

#### Inclusion criteria

(1) Skin lesion suspected of being CMM planned for surgical removal following standard guidelines. (2) At least 18 years of age. (3) Written informed consent for participation in the study.

#### Exclusion criteria

(1) Longest lesion diameter shorter than 3 mm. (2) Lesion with bleeding, ulceration, or serous exudation. (3) Use of systemic steroids or topical steroids on the lesion within 30 days before tape stripping. (4) Allergy to tape. (5) Pregnancy or breastfeeding.

### Participant recruitment and enrollment

Patients are recruited at the Department of Dermatology or the Department of Plastic Surgery and Burns Treatment at Copenhagen University Hospital. Patients are referred to these departments with skin lesions suspected of being CMM. The lesions are examined, and it is decided whether surgical removal is indicated. Patients who need surgical removal of a suspicious lesion are screened according to the eligibility criteria. Eligible patients are given oral and written information about the study. Subsequently, informed written consent for participation is obtained. Sample and data collection are obtained just before the surgical excision. The surgical excision is performed according to standard guidelines without any delay, see Fig 1.

### Sample and data collection

Data are collected at Copenhagen University Hospital, the Department of Dermatology or the Department of Plastic Surgery and Burns Treatment. The lesions’ diameters and locations are noted, as are the patients’ unique Danish personal identification numbers [17], which makes it possible to link the definitive histopathologic diagnosis to the results of the RNA analysis. The number also holds age information.

**Fig 2:**
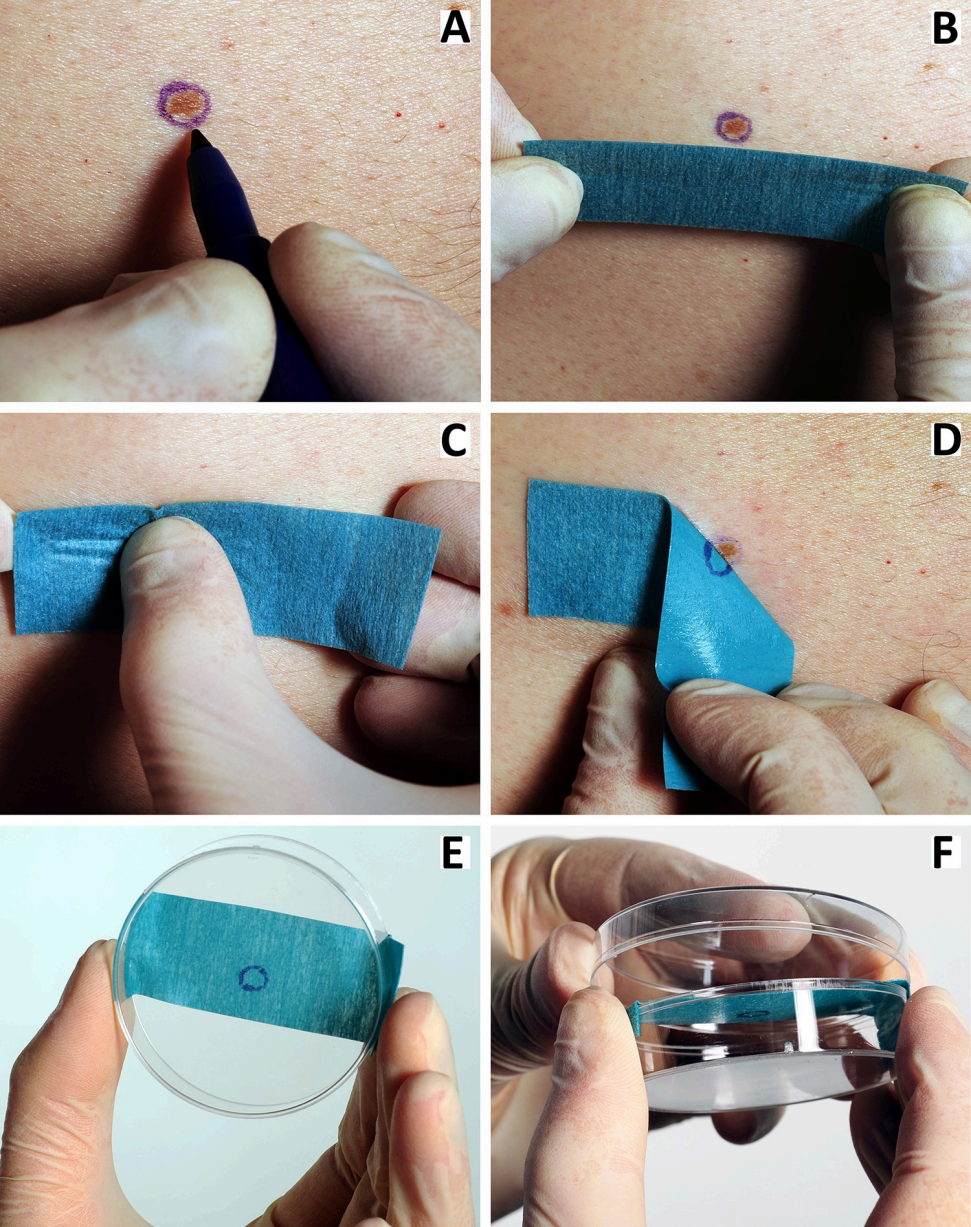
Tape stripping. A sequence of pictures illustrating the tape stripping procedure. First, a line is drawn around the lesion with a pen (**A**). After approximately 15 seconds, a tape strip is placed on the lesion, and the investigator massages the tape gently against the lesion for 20 seconds using the thumb (**B, C**). The tape strip is then removed from the skin (**D**) and transferred to the rim of a Petri dish with the harvested RNA facing down, not touching the sides of the dish (**E**). The lid is mounted to fix the tape and protect against contamination (**F**). The sample is handled with gloves to avoid contamination with RNA from the investigator.

The tape stripping procedure is shown in Fig 2. First, the border of the suspicious lesion is marked by a sterile surgical skin marking pen (Viscot Medical LLC, East Hanover, NJ, USA) (Fig 2A). Then a piece of sterile hospital barrier tape (Mölnlycke Health Care, Göteborg, Sweden) is cut out. The piece must be approximately 7 cm long, making it long enough to reach from one side to the other side of a 5 cm petri dish. The width of the tape is adapted to the size of the lesion. The tape is placed upon the skin covering the marked lesion and gently massaged for 20 seconds. The skin is not stretched during sampling (Fig 2B and 2C). The tape is gently stripped from the skin, leaving a mark on the tape corresponding to the marked perimeter of the lesion (Fig 2D). The tape is then suspended across the rim of the open bottom part of a cylindrical sterile Petri dish so that the tape adheres to the outer sides of the dish walls. The side with biological material faces down and away from the open top and remains untouched (Fig 2E). The lid is mounted to fix the tape and protect against contamination (Fig 2F). Tape stripping is performed twice to increase the amount of sample material. The tape is placed at the same skin site both times. Each piece of tape is placed in its own Petri dish. The Petri dishes are stored at room temperature in a paper envelope to avoid sunlight until RNA analysis. The packaging is air permeable to allow the samples to dry. The samples are handled with gloves to avoid contamination with RNA from the investigators.

### Gene expression analysis

Gene expression is investigated through the analysis of RNA profiles on the tape strips using quantitative real-time polymerase chain reaction (qRT-PCR). It will be performed blinded by pseudo-anonymization at the Section of Forensic Genetics, Department of Forensic Medicine, Faculty of Health and Medical Sciences, University of Copenhagen. All laboratory personnel are blinded to the histopathological diagnoses. Several aspects of the methods have been optimized, including probe concentrations, temperatures, and timing to achieve as low threshold cycle values as possible. We have established the following method. First, the tape outside the pen mark is removed using a scalpel. No further cuts are made to the remaining tape strip. RNA is extracted from the tape with the miRNeasy Micro Kit (Qiagen Denmark, Copenhagen, Denmark) following the manufacturer’s recommendations (RNeasy Mini Handbook, Protocol: Purification of Total RNA, including miRNA, from Animal Cells, 03/21). No additional steps are performed to remove the biological material from the tape. A sample from one lesion consists of two tape strips. To each of the two tape strips 700 μl QIAzol Lysis Reagent (Qiagen) is added. After addition of chloroform (140 μl per tape strip, Amresco, Biotechnology Grade, Saint Neots, United Kingdom), the suspensions are divided into the aqueous and organic phases by centrifugation. The tape strips are kept in the tubes for the duration of the phase separation when the aqueous phase from each of the two tapes are pooled on one column. The RNA is eluted in 45 μl RNase-free water. The resulting liquid is named the RNA solution and is distributed for examination of the 11 genes.

The isolated RNA is then used for cDNA synthesis. Since the expression of the 11 genes differs, but is generally low, each quantification of a gene will be carried out in singleplex, except for the two housekeeping genes that are investigated in the same reaction. For each RNA cDNA synthesis and expression, the analysis is performed in a one-step reaction using TaqMan probes and the EXPRESS One-Step SuperScript qRT-PCR Universal kit (Thermo Fisher Scientific, Waltham, MA, USA) following the manufacturer’s recommendations, but with half the volume: 12.5 μl EXPRESS qPCR SuperMix Universal, 0.05 μl ROX Reference Dye (25 μM), 2.5 μl EXPRESS SuperScript Mix for One-Step qPCR, and 5.7 μl RNAse-free water, 1.25 μl TaqMan probe, and an RNA solution volume depending on the gene analyzed. The probes and corresponding volumes of the RNA solutions are listed in Table 2. At each investigation of patient samples, a negative control is concurrently examined. The negative control sample consists of a circular piece of tape with a diameter of 1 cm that has not been in contact with skin. The expression of each of the 11 genes is measured using an Applied Biosystem 7500 Fast Real-Time PCR (Thermo Fisher Scientific) with the following setup: 15 min at 50°C, 2 min at 95°C, followed by 45 cycles of 95°C for 15 s and 60°C for 1 min. The gene expression levels will be combined into a binary outcome (see Statistics section). Positive indicates CMM, negative indicates no CMM.

### Histopathological diagnosis

The definitive diagnosis of each lesion is made by routine histopathology by a pathologist at the Department of Pathology, Copenhagen University Hospital, blinded for the RNA analysis. Lesions are classified as benign nevi, CMMs, or another diagnosis. CMMs are subclassified as: superficial spreading, nodular, lentigo maligna, acral lentiginous, amelanotic, spitzoid, desmoplastic, or pigment synthesizing [18]. The thickness of each CMM is measured in millimeters from the granular layer of the epidermis to the deepest malignant cell in the dermis or subcutaneous tissue [19].

### Statistics

Demographic and clinical data will be summarized using standard descriptive statistics.

The main outcome variables are the results of the RNA analysis and the histopathological diagnosis which, combined, will provide the sensitivity and specificity of the RNA test. For our purposes, test sensitivity is the more important factor. Consequently, we chose our sample size to give reasonable precision in estimating sensitivity based on power calculations presented by Buderer [20]. With an expected sensitivity of 95% (*S*), a type I error rate of 5% (*α*), a confidence interval size of 10% (*W*), and an expected prevalence of CMM in subjects of 10% (*P*), we need 183 patients with complete test results. $N=\frac{Z_{\alpha }^{2}\times S\times (1-S)}{W^{2}\times P}=\frac{1.96^{2}\times 0.95\times (1-0.95)}{0.10^{2}\times 0.10}=183$. To compensate for any dropout, approximately 200 individuals with a lesion suspected of being CMM and requiring surgical removal will be included.

The gene expression levels will be combined into a binary outcome. Positive indicates CMM. Negative indicates the absence of CMM. First, a binary logistic regression using a backward stepwise method will be performed including all 11 RNA parameters using results from all samples to determine significant parameters. Only parameters that are statistically significant after completing the backward regression will be used in the final RNA test. We will plot a receiver operating characteristic (RUC) curve and calculate the area under the RUC curve to summarize the trade-off between the true positive and false positive rates. Afterward, we plan to evaluate the model of the established RNA test using leave-one-out cross-validation. The positive and negative predictive values will be reported. In addition, the study will investigate the impact on the RNA test of the patient’s age, lesion diameter, skin area involved, and storage time of the tape before analysis. Subsequently, we will search for other possible confounders.

P-values below 0.05 will be considered significant. All analyses will be done in IBM SPSS statistics (IBM, Armonk, NY, USA).

### Trial registration

The project was approved by the Committee on Health Research Ethics of the Capital Region of Denmark (approval number: H-15010559) and by the Danish Data Protection Agency (journal number: BFH-2015-065).

## Discussion

The study aims to validate if an RNA test can provide clinicians with valuable additional information when deciding whether to excise a clinically suspicious lesion or not [21]. This will have the potential to reduce unnecessary surgical procedures and thus decrease patient morbidity and costs. The RNA test may be particularly useful in cosmetically and functionally sensitive areas or in patients with an increased risk of impaired wound healing or keloid scars.

It is a serious concern that a sensitivity of about 91% to 95%, as found in the previous RNA test based on two genes [10, 12, 13], may result in missed CMMs. A rule-out test of CMM must have a very high sensitivity, close to 100%, to be generally accepted. Therefore, this protocol includes expression analysis of 9 disease related genes and two housekeeping genes (listed in Table 2). The final diagnosis of cutaneous melanocytic lesions relies on the pathologist’s visual valuation of the excised lesion. Studies have found a substantial interobserver variability in the interpretation of CMM lesions [22, 23]. Hence it is impossible for the RNA test to have a sensitivity of 100% as the pathologists’ diagnoses are not 100% accurate.

Only the outermost layers of the stratum corneum are removed during the tape stripping procedure. This does not impact the subsequent histological examination of the underlying epidermis [8, 24, 25]. No living cells or nuclear material are collected by the tape strip method. At first, it was surprising that RNA was found on tape strips from the stratum corneum of both normal skin, CMMs, and other skin diseases. Subsequently, the findings have been confirmed by several studies [8, 26–28]. Investigations of tape strips have also been widely used in dermatology to determine protein, lipid, and microbial expression noninvasively [29, 30].

We have established the method described in the Methods section by analyzing 118 tape strips after normal human skin sampling in our laboratory at the Section of Forensic Genetics, Department of Forensic Medicine, Faculty of Health and Medical Sciences, University of Copenhagen. Four genes (*B2M*, *CLTC*, *GAPDH*, and *NAGPA*) were investigated as potential housekeeping genes. The overall expression level of *NAGPA* was low compared to those of the other candidates, and *NAGPA* could not be detected in several samples. Consequently, *NAGPA* is not used as a housekeeping gene in the present protocol. We have measured the levels of the chosen RNAs (see Table 2) from normal skin sampled with tape strips from circular areas. As there was abundant material when sampling areas with diameters down to 4 mm in the pilot study, we decided to include lesions with diameters of 3 mm or more in the forthcoming clinical trial. It was possible to obtain the results without RNA amplification and thus reduce material loss, noise, and bias. We will carry out our project only with qRT-PCR amplification of cDNA. The final project will focus solely on measuring the expression levels of the 11 genes. The investigated housekeeping genes (see Table 2) will be used to assess the RNA quality of the sample.

To make the test clinically useable, tape strips were stored in a dark and dry place at room temperature for up to 14 days. The pilot study showed that the material could be stored under these conditions improving the ease of shipment from clinic to laboratory. The study of diameter and storage options was performed as a pilot study and must be confirmed in the final study with this protocol.

Likewise, in another pilot study, we identified the RNAs in 54 formalin-fixed paraffin-embedded samples, including 24 benign nevi and 30 CMMs. In these 54 samples, the nine disease-related genes were differently expressed in CMMs compared to nevi. The two housekeeping genes *CLTC* and *GAPDH* were equally expressed. *B2M* that is usually considered a housekeeping gene with similar expression in many tissues was differently expressed in CMMs compared to nevi. Consequently, *B2M* was excluded as a housekeeping gene. On this basis, we will initiate the study presented in this protocol to investigate the RNAs presented in Table 2 from lesions suspected of being CMM.

Our study population consists of patients referred for surgical removal of suspicious lesions. Consequently, the study will provide information about the sensitivity and specificity of the RNA test used in this clinical situation. In the future, it could also be interesting to examine how the test performs in a primary care setting or in clinics that follow individuals with numerous atypical nevi showing some of the clinical features of melanoma [31].
